# Cancer incidence and mortality trends in Australian adolescents and young adults, 1982–2007

**DOI:** 10.1186/1471-2407-12-151

**Published:** 2012-04-20

**Authors:** Fatima A Haggar, David B Preen, Gavin Pereira, Cashel DJ Holman, Kristjana Einarsdottir

**Affiliations:** 1 School of Population Health, Centre for Health Services Research, The University of Western Australia, Crawley, Australia; 2 The Department of Surgery, The Ottawa Hospital Research Institute, The University of Ottawa, Ottawa, Canada; 3 Telethon Institute for Child Health Research, Centre for Child Health Research, The University of Western Australia, Subiaco, Australia

**Keywords:** Cancer, Epidemiology, Adolescents, Young adults, Incidence, Mortality, Trends, Population-based

## Abstract

**Background:**

Increasing incidence and lack of survival improvement in adolescents and young adults (AYAs) with cancer have led to increased awareness of the cancer burden in this population. The objective of this study was to describe overall and type-specific cancer incidence and mortality trends among AYAs in Western Australia from 1982–2007.

**Methods:**

Age–adjusted incidence and mortality rates were calculated for all malignancies combined and for each of the most common diagnostic groups, using five-year age–specific rates. Joinpoint regression analysis was used to derive annual percentage changes (APC) for incidence and mortality rates.

**Results:**

The annual incidence rate for all cancers combined increased in males from 1982 until 2000 (APC = 1.5%, 95%CI: 0.9%; 2.1%) and then plateaued, whilst rates for females remained stable across the study period (APC = −0.1%; 95%CI: −0.2%; 0.4%) across the study period. For males, significant incidence rate increases were observed for germ cell tumors, lymphoblastic leukemia and thyroid cancer. In females, the incidence of Hodgkin’s lymphoma, colorectal and breast cancers increased. Significant incidence rate reductions were noted for cervical, central nervous system and lung cancers. Mortality rates for all cancers combined decreased from 1982 to 2005 for both males (APC = −2.6%, 95%CI:−3.3%;−2.0%) and females (APC = −4.6%, 95%CI:−5.1%;−4.1%). With the exception of bone sarcoma and lung cancer in females, mortality rates for specific cancer types decreased significantly for both sexes during the study period.

**Conclusions:**

Incidence of certain AYA cancers increased, whilst it decreased for others. Mortality rates decreased for most cancers, with the largest improvement observed for breast carcinomas. Further research is needed to identify the reasons for the increasing incidence of certain cancers.

## Background

Cancers in adolescents and young adults (AYAs), aged 15–39 years, account for less than 10% of all new cancer diagnoses in developed countries [1]. The most commonly occurring malignancies in this age group differ markedly from those in older adults and younger children [2,3]. In children (<15 years), embryonal tumors represent the most prevalent neoplasms, whereas the most common cancers in older adults (>39 years) are epithelial malignancies of the lung, prostate, breast and colorectum [2]. In AYAs, nearly all invasive cancers are accounted for by lymphomas, leukemias, germ cell tumors, melanoma and carcinomas [4,5].

Recent major advances in treatment and coordinated international research efforts have led to remarkable improvements in childhood cancer outcomes. However, AYAs, while having a higher and increasing incidence of cancer, have not benefited from similar improvements. During the past years, this deficit has become the subject of international focus and initiatives [2,6] but in Australia, few empirical reports have specifically focused on cancer outcomes among AYAs. Herein, we provide data on recent cancer incidence and mortality rates, and a detailed analysis of long–term trends for the most prevalent malignancies among AYAs, using routinely-collected administrative health data in Western Australia (WA) during the period 1982–2007.

## Methods

A retrospective cohort study of AYA cancer incidence and mortality trend was conducted using whole-population, health record linkage in WA. Incident cases included all malignant neoplasms registered from January 1, 1982 to December 31, 2007. Patient death date used to calculate mortality trend was restricted to December 31, 2005. Cases were restricted to those in individuals aged 15–39 years at diagnosis. Notifications of all malignancies, including *in situ* and malignant neoplasms and excluding non-melanoma skin cancer, have been a statutory requirement since 1981 in WA. Cancer registrations include information on basic demographic data (date of birth, sex, Aboriginality, area of residence) and tumor data (date of diagnosis, tumor site, morphology, behaviour, grade, basis of diagnosis, information on subsequent primary malignancies) and vital status. Active data follow-up of all patients was performed by the WA Data Linkage Service (WADLS) through linkage of the Cancer Registry, the Mortality Register, and the Hospital Morbidity Data System, which contains information on all hospital separations within WA. Anonymised linked records from the Cancer Registry, the Mortality Register, and the Hospital Morbidity Data System (containing information on all hospital separations within WA) were provided by the WADLS. Malignancies were classified according to histological origin as described in the 3^rd^ edition of the *International Classification of Diseases for Oncology* and further grouped according to the most prevalent cancer types based on the Surveillance, Epidemiology, and End Results Program (SEER) AYA cancer diagnostic groups (Table 1). The groups were based on the AYA classification scheme which was developed to better define the major cancer sites that affect individuals between 15 and 39 years of age [7].

**Table 1:** Number of cases, sex ratio, and International Classification of Diseases for Oncology topography and histology codes

**Diagnostic Group**	***n*(%)**	**M:F**
**Leukaemia**	**444 (3.6)**	**1.4**
ALL	120 (1.0)	1.7
AML & CML	276 (2.3)	1.2
Other	48 (0.4)	2.5
**Lymphoma**	**907 (7.4)**	**1.3**
NHL	472 (3.9)	1.6
HL	435 (3.5)	1.0
**CNS Tumour**	**406 (3.3)**	**1.3**
**Bone**	**183 (1.5)**	**1.4**
**Soft Tissue**	**323 (2.6)**	**1.4**
**Germ Cell**	**960 (7.8)**	**10.5**
Gonadal	875 (7.1)	24.0
Non−gonadal	85 (0.7)	1.4
**Melanoma**	**3551 (29.0)**	**0.9**
**Carcinoma**	**5012 (40.9)**	**0.4**
Thyroid	640 (5.2)	0.3
Lip & oral cavity	515 (4.2)	3.6
Gonads	107 (0.9)	0.01
Lung	129 (1.1)	1.1
Breast	1440 (11.8)	na
Cervix	782 (6.4)	na
Colorectum	500 (4.1)	1.0
Other	899 (7.3)	2.2
**Other**	**452 (3.7)**	0.6
**Total**	**12238**	**0.7**

*CNS* central nervous system; *ALL* acute lymphocytic leukaemia; *AML* and *CML* acute and chronic myeloid leukaemia;

*HD* Hodgkin’s disease; *NHL* Non−Hodgkin’s lymphoma; M:F: Sex ratio, *na* not applicable.

### Data analysis

Five-year age−adjusted incidence and mortality rates were calculated by sex and SEER AYA diagnostics groups. Rates were adjusted by direct standardization against the five−year age distribution of the standard Australian population in 2001. Annual population estimates used in the calculation of the rates were obtained from the Australian Bureau of Statistics. Analyses were performed for all malignancies combined and for diagnostic groups and subtypes separately. Joinpoint regression analysis was used to identify points at which statistically significant changes in temporal trend occurred. The annual percentage change (APC) in each joinpoint segment is the rate of change in a cancer rate per year in a given time frame. Changes in rates included a shift in the magnitude or a change in the direction of the rate. A negative APC indicates a decreasing trend whereas a positive APC indicates an increasing trend. Joinpoint analyses were performed using the Joinpoint Regression Program (3.4) [8]. Subtypes of certain diagnostic groups were excluded from joinpoint regressions because there were too few cases to allow for a trend analysis. Incidence and mortality rates and standard errors were calculated using SAS 9.2.

Ethics approval for this study was obtained from the University of Western Australia Research Ethics Committees (reference number: RA/4/1/2228).

## Results

There were 12238 incident cases of malignant neoplasms reported from 1982–2007, among AYAs aged 15–39 years. Table 1 presents the histological distribution of the tumours. Detailed data on the AYA classification groups and histological codes are provided as Additional file 1: Table S1.

### All malignancies combined

**Figure 1:**
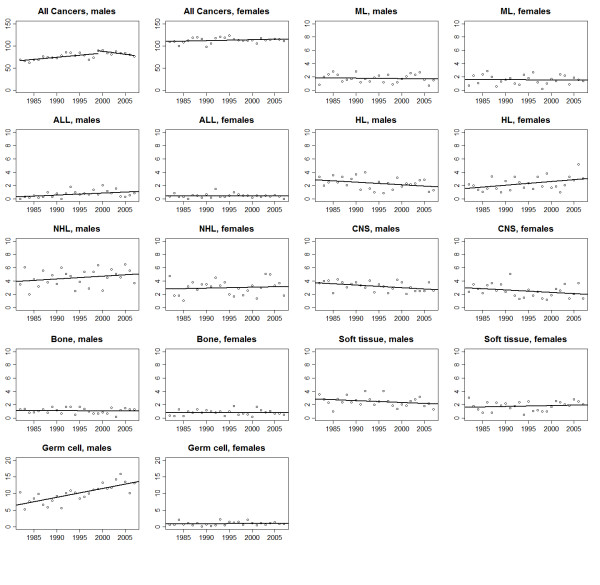
**Trends in age−adjusted incidence for all malignancies combined and selected diagnostic groups for adolescents and young adults of ages 15–39 years, 1982–2007**^**a**^**.**^a^ dots represent observed rates and solid lines represent the (LOWESS) smoothed trend.

**Figure 2:**
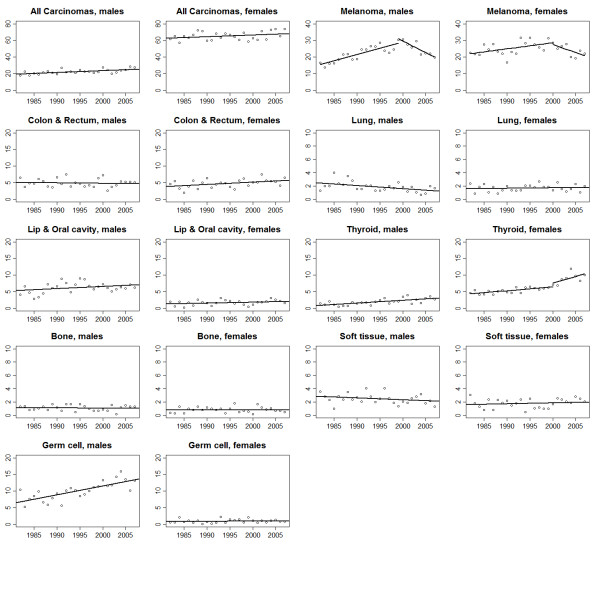
Trends in age−adjusted incidence for all malignancies combined and selected diagnostic groups for adolescents and young adults of ages of 15–39 years, 1982–2007.

**Figure 3:**
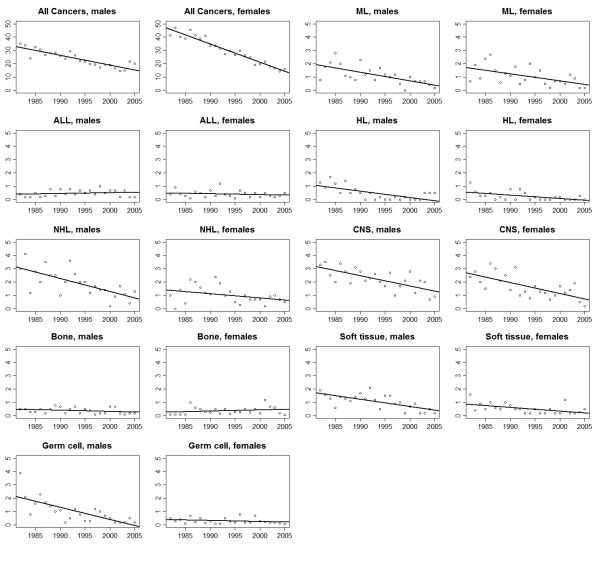
Trends in age−adjusted mortality for all malignancies combined and selected diagnostic groups for adolescents and young adults of ages 15–39 years, 1982–2005.

**Figure 4:**
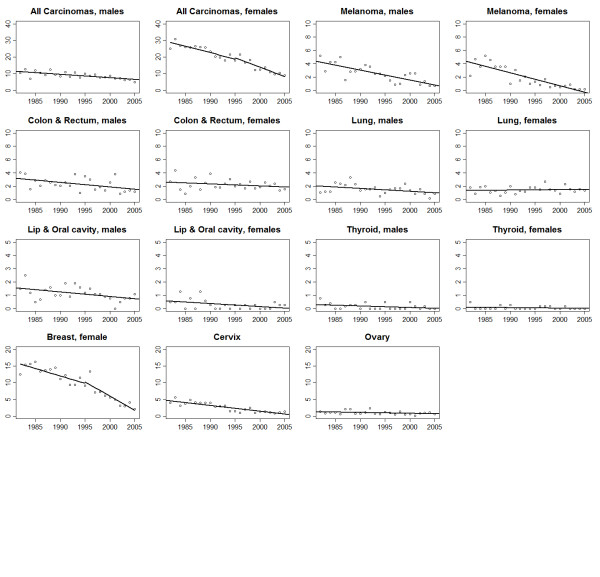
Trends in age−adjusted mortality for all malignancies combined and selected diagnostic groups for adolescents and young adults of ages 15–39 years, 1982–2005.

Trends in age−adjusted rates and the results of the joinpoint analysis for all malignancies combined and for the individual cancer types are shown in Figures 1, 2, 3 and 4 and Tables 2 &3. The overall incidence of malignancies in males significantly increased between 1982 and 2000 (APC = 1.5%), after which there was no significant change in the rates. In contrast, incidence rates for females remained stable throughout the study period. Mortality significantly decreased, during the study period, for both males (APC = −2.6%) and females (APC = −4.6%).

**Table 2:** Joinpoint analysis of age−adjusted incidence rates per 100 000, AYAs aged 15–39 years in Western Australia, 1982–2007

	**Incidence rates, Males**				**Incidence rates , Females**			
	**Trend 1**		**Trend 2**		**Trend 1**		**Trend 2**	
**Diagnostic Groups**	**Period**	**APC 95% CI**^**c**^	**Period**	**APC 95% CI**	**Period**	**APC 95% CI**	**Period**	**APC 95% CI**
**All Tumours**	82 − 00	1.5* ( 0.9; 2.1)	00 − 07	−1.4*(-3.4;0.7)	82 − 07	−0.1* (-0.2; 0.4)		
**Leukaemia**	82 − 07	0.8 *(-0.7; 2.4)			82 − 07	0.0* (-2.1; 2.1)		
Myeloid	82 − 07	−0.3* (-2.1; 1.6)			82 − 07	−0.4* (-2.6; 2.0)		
Lymphocytic	82 − 07	3.6* ( 0.1; 7.2)			82 − 07	−1.5* (-4.5; 1.6)		
**Lymphoma**	82 − 07	0.0* (-1.2; 1.3)			82 − 07	1.4* (-0.3; 3.1)		
NHL	82 − 07	0.9* (-0.6; 2.4)			82 − 07	0.5* (-1.5; 2.5)		
HL	82 − 07	−1.5* (-3.3; 0.3)			82 − 07	2.6* ( 0.6; 4.7)		
**CNS**	82 − 07	−1.2* (-2.3; 0.1)			82 − 07	−1.2* (-3.2; 0.8)		
**Bone Tumor**	82 − 07	−0.1* (-2.0; 1.9)			82 − 07	0.0* (-2.7; 2.8)		
**Soft Tissue**	82 − 07	−1.2* (-2.9; 0.4)			82 − 07	0.8* (-1.1; 2.7)		
**Germ Cell**	82 − 07	2.6* ( 1.6; 3.6)			82 − 07	0.1* (-2.7; 3.0)		
Gonadal	82 − 07	2.6* ( 1.8; 3.9)			82 − 07	−0.1* (-3.6; 3.4)		
Non-gonadal	82 − 07	−0.6* (-3.7; 2.5)			82 − 07	0.2* (-3.6; 4.2)		
**Melanoma**	82 − 00	3.5* ( 2.3; 4.6)	00 − 07	−5.3*(-9.1;-1.4)	82 − 99	1.6* ( 0.0; 3.1)	99 − 07	−3.8*(-7.7; 0.3)
**Carcinoma**	82 − 07	1.0* ( 0.4; 1.5)			82 − 07	0.3* (-0.1; 0.7)		
Breast	na	na			82 − 07	0.5* ( 0.0; 0.9)		
Colon & Rectum	82 − 07	−0.4* (-1.7; 1.0)			82 − 07	1.4* ( 0.1; 2.7)		
Ovary	na	na			82 − 07	−0.3* (-2.3; 1.9)		
Lung	82 − 07	−2.6* (-4.3;-0.9)			82 − 07	0.4* (-1.3; 2.0)		
Cervix Uteri	na	na			82 − 07	−2.7* (-3.5;-1.8)		
Thyroid	82 − 07	4.0* ( 2.0; 6.1)			82 − 00	2.1* ( 0.9; 3.2)	00 − 07	13.8*(-0.6;30.3)
Lip & Oral Cavity	82 − 07	0.6* (-0.7; 2.0)			82 − 07	1.1* (-1.1;-3.3)		

* The annual percentage change (APC) is statistically significantly different from 0 (*p* < 0.05).

^a^ Effective sample size less than 10.

^c^95% CI, 95% confidence interval.

**Table 3:** Joinpoint analysis of age-adjusted mortality rates per 100 000, AYAs aged 15–39 years in Western Australia, 1982–2005

	**Mortality rates, Males**		**Mortality rates, Females**			
	**Trend 1**		**Trend 1**		**Trend 2**	
**Diagnostic Groups**	**Period**	**APC 95% CI**	**Period**	**APC 95% CI**	**Period**	**APC 95% CI**
**All Tumours**	82 − 05	−2.6* (-3.3; -2.0)	82 − 05	−4.6* (-5.1; -4.1)		
**Leukaemia**	82 − 05	−3.6* (-5.9; -1.2)	82 − 05	−3.8* (-6.1; -1.5)		
Myeloid	82 − 05	−5.6* (-8.0; -3.1)	82 − 05	−5.0* (-8.3; -1.6)		
Lymphocytic	82 − 05	1.6 (-1.5; 4.8)	82 − 05	−2.2 (-5.3; -0.9)		
**Lymphoma**^a^	82 − 05	−5.4* (-7.4; -3.4)	82 − 05	−4.9* (-7.7; -2.0)		
NHL	82 − 05	−4.8* (-7.1; -2.5)	82 − 05	−4.5* (-7.3; -1.7)		
**CNS**	82 − 05	−3.1* (-4.8; -1.4)	82 − 05	−4.6* (-6.9; -2.1)		
**Bone**	82 − 05	−1.8 (-5.0; 1.5)	82 − 05	4.3* (-0.4; 9.2)		
**Soft Tissue**	82 − 05	−4.7* (-7.6; 1.8)	82 − 05	−3.7* (-7.0; -0.2)		
**Germ Cell**^a^	82 − 05	−8.2* (-10.5;-5.8)	82 − 05	−1.3 (-6.1; 3.6)		
Gonadal	82 − 05	−7.7* (-10.3;-5.1)				
**Melanoma**	82 − 05	−5.5* (-7.5; -3.4)	82 − 05	−10.9* (-13.5;-8.2)		
**Carcinoma**^a^	82 − 05	−2.4* (-3.4; -1.5)	82 − 96	−3.0* (-4.2; -1.7)	96 − 05	−8.2* (-10.7; -5.5)
Breast	na	na	82 − 96	−3.0* (-4.2; -1.3)	96 − 05	−14.4* (-18.4; -9.9)
Colon & Rectum	82 − 05	−2.1* (-4.0; -0.1)	82 − 05	−2.3* (-3.7;-0.8)		
Ovary	na	na	82 − 05	−2.6 (-5.5; -0.5)		
Lung	82 − 05	−2.8* (-5.2; -0.3)	82 − 05	−0.9* (-1.3; 3.1)		
Cervix Uteri	na	na	82 − 05	−6.8* (-8.2; -5.3)		
Lip & Oral Cavity	82 − 05	−3.4* (-5.5; -1.3)				

* The annual percentage change (APC) is statistically significantly different from 0 (*p* < 0.05).

^a^ Data for HL, non-gonadal germ cells and thyroid cancers and lip/oral in females were not reported due to low effective sample size less than 95% CI, 95% confidence interval.

### Leukemias and lymphomas

The incidence of acute lymphoblastic leukaemia (ALL) increased significantly among males (APC = 3.6%), and that of Hodgkin’s lymphoma (HL) increased (APC = 2.6%) among females. No evidence of change in incidence was observed for other haematological cancers.

A significant downward trend in myeloid leukaemia mortality was evident (males, APC = −5.6%; females, APC = −5.0%). A significant downward trend in Non-Hodgkin Lymphoma (NHL) mortality was also observed among both males (APC = −4.8%) and females (APC = −4.5%).

### CNS, bone and soft tissue sarcomas & germ cell tumors

For all CNS tumours combined a modest (but statistically significant) decrease in incidence rates was observed in males (APC = −1.2%) with no trend evident among females. Male germ cell tumours, comprising predominately (approximately 94%) gonadal tumors, also increased (APC = 2.6%). No changes in the incidence of bone tumours or soft tissue tumours in either sex, or of female germ cell tumours were evident, although this is likely due to the smaller number of cases.

A significant decline in mortality from CNS tumours was observed from 1982-2005 for males (APC = −3.1%) and between 1982 and 2005 for females (APC = −4.6%). Soft tissue sarcoma mortality decreased among females (APC = −3.7%). No evidence of change in mortality from soft tissues sarcoma was observed for the males.

### Melanoma and carcinomas

Incidence of melanoma significantly increased from 1982 until 1999 for males (APC = 3.5%) and until 2000 for females (APC = 1.6%). Afterwards, the incidence of melanoma declined significantly for both males (APC = −5.3%) and females (APC = −3.8%). A small but significant increase in incidence was observed for breast carcinomas (APC = 0.5%). Cervical carcinoma incidence (APC = −2.7%) also decreased significantly. Thyroid carcinoma incidence increased significantly for both sexes (males, APC = 4.0%; females, APC = 2.1%). Incidence rates increased significantly for colorectal carcinoma (APC = 1.4%) among females only and lung carcinoma incidence significantly decreased for males only (APC = −2.6%).

Melanoma mortality decreased from 1982-2005 for both sexes (male, APC = −5.5%; females, APC = −10.9%). Mortality rates for all carcinomas combined decreased for males (APC = −2.4%). In females, incidence (−3.0%) was similar to males from 1982 − 96 but a steeper decline in mortality was observed thereafter (APC = −8.2%). This trend was likely driven by breast cancer mortality, which significantly decreased from 1982–1996 (APC = −2.9%) and was followed by another more rapid significant decrease thereafter (APC = −14.4%). Mortality from ovarian cancer (APC = −2.6%) and cervical cancer (APC = −6.8%) cancer decreased significantly. Significant reductions in mortality rates were observed for colorectal carcinoma in both males (APC = −2.1%) and females (APC = −2.3%). Lung carcinoma mortality significantly decreased for males only (APC = −2.8%).

## Discussion

Improvements in modern treatments and diagnosis has led to reductions in incidence and mortality attributed to many neoplasms, including those that commonly occur among AYAs [5]. However, incidence is still increasing for some cancers that commonly occur among AYAs and decline in mortality has lagged for certain cancers. This is the first study to have comprehensively investigated cancer incidence and mortality trends at the whole population level for a broad range of cancers that commonly occur among Australian AYAs aged 15–39 years using the adolescent and young classification scheme.

Increases in incidence rates were observed for testicular cancer, thyroid cancer, and ALL among male AYAs, whereas in young females, the incidence of HL, breast, and colorectal cancers increased over the 26-year study period. Decreases in the incidence of lung cancer and CNS tumors were observed among males only, cervical cancer among females, and melanoma for both the sexes. Between 1982-2005, cancer mortality rates decreased for both sexes, although considerable disparity existed between males and females by cancer type.

Despite expanding knowledge on many AYAs cancers, the reasons for the increasing incidence of some cancers are still poorly understood, particularly for testicular cancer [9]. Known risks factors for testicular cancer include cryptorchidism, testicular atrophy and maternal exposures [10,11]. Exposure to ionizing radiation, particularly in childhood, may influence the incidence of thyroid carcinoma later in life [12]. However, the sex differential observed among AYAs in this study is not clear, although it may imply a specific susceptibility gene hormone receptor in the pathogenesis of thyroid carcinomas or possibly due to a greater medical surveillance in young women. Some of the increase in the incidence of thyroid cancer may be also explained by the use of improved diagnostic tests, although diagnostic scrutiny alone is unlikely to explain the trends observed in this study. Decreasing exposures to infections in childhood and thereby increased susceptibility later in life may play a role in the occurrence of both ALL and HL in adolescence and young adults. In fact, Epstein Barr virus, in particular, may play a role in the incidence of a substantial proportion of HL cases [13].

There was a small but significant increase in breast cancer incidence. This may be a result of recent improvements in diagnostic imaging of the dense fibroglandular breast in young women and adjuvant chemotherapy and radiation therapy. Other potential contributing factors may include use of oral contraceptives and changes in the fertility patterns [14]. Fortunately, breast cancer mortality among young WA women decreased over the same period. In fact, this group experienced the greatest decrease in mortality in this study, particularly post 1996. This is likely due advances in newer imaging techniques, such as magnetic resonance imaging (MRI), that can detect tumors previously occult because of the dense fibroglandular breast in young women. The recent reduction in the financial barrier through a Medicare rebate for MRI fee for young women <50 years of age and at high risk of breast cancer has the potential to further decrease mortality in this population.

The overwhelming majority of lung cancers in AYAs are caused by tobacco smoking. Tobacco control is currently a priority in Australia, with WA seen as the national leader in this area [15]. Smoking prevention and cessation programs are aimed not only at the general population, but are increasingly targeting young people. However, while there has been some recent evidence of a decreasing trend in both incidence and mortality among men [16], the situation in women, particularly young women, is a cause for concern. The present study results indicate that young women are not experiencing the same decline in lung cancer incidence as young men, which is consistent with known disparity of historical smoking uptake and cessation trends between genders [17,18]. Nonetheless, it is expected that recent declines in female smoking rates would eventually result in a decline in the future incidence and mortality for AYA females [19].

Colorectal cancer is uncommon among younger AYAs but rises substantially after the age of 25 years [20]. The incidence of colorectal cancer increased for young females in our study, but not in males. Yet, hereditary predisposition, the main risk factor in this age group is expected to be similar in both sexes [21]. Sex hormones have immuno-modulatory effects and may differentially affect the incidence of colorectal cancer in AYAs [22-24]. However, the observed trend in incidence among females in our study is more likely to be attributed to environmental factors, such as changes in nutrition, smoking and alcohol consumption [21], although these factors could not be evaluated in our study [21]. Overall, mortality due to colorectal cancer has decreased substantially in the last decades. Recognition of familial colorectal cancer syndromes and refinement of diagnostic techniques are likely to have contributed to reductions in mortality among AYAs.

Trends in incidence and mortality of both cervical cancer and melanoma are largely influenced by earlier cancer detection and improved treatments. Cervical cancer screening to detect potentially pre-cancerous changes which are usually caused by sexually transmitted human papillomaviruses (HPV) infection is likely responsible for the decreases in incidence and deaths observed in our study. The recent implementation of the HPV immunization program in Australia, in 2007, is likely to further reduce the burden of cervical cancer. In WA, the spread of independent skin cancer clinics might have initially increased the incidence of cutaneous melanomas as result of increased detection of indolent cases through increased surveillance [25]. The rise seems to have reversed more recently, possibly as a result of changes in recreational behavior and increased protection from sun exposure [26]. Continued skin awareness campaigns are likely to further decrease incidence and mortality due to melanomas.

The overall pattern of increasing incidence [27-30] and decreasing mortality [31,32] of AYA cancers reported in this study approximates that observed in other developed countries, including in the United States, Canada and some western European nations. Increasing incidence of specific cancers such as thyroid [33-35], testicular [36,37] observed in this study population appears to be consistent with results from other AYA populations. The emerging lung cancer epidemic observed among females in our study is also consistent with other studies from several developed countries [38-41]. Cervical cancer incidence and mortality rates continued to decrease among AYAs in WA and other developed countries [42,43], although declines seem to be moderating or plateauing in some populations [44,45].

A strength of this study is its use of routinely-collected, whole-population data from the WADLS, which has undergone extensive validation with false-positives and false-negatives shown to be <1% [46]. Using these data, we investigated incidence and mortality trends over a 26-year period for AYAs aged between 15-39 years, and this is the first comprehensive analysis of cancer incidence and mortality trends among for a broad range of cancers that commonly occur among AYAs in Australia. Although WA’s population of 2.3 million is not very large, WA population is representative of the rest of Australia; it is closest to the average of the eight Australian states and territories with respect to: median age; distribution of sex, people living in remote areas, income, per capita health expenditure and hospital bed supply. In addition, the relatively low and stable overseas and internal migration (~2%) of the WA population has the advantage of reduced loss to follow-up. These characteristics of WA and the completeness of our data strengthened our study.

However, a number of challenges exist, particularly in the interpretation of results for rare cancers. Random fluctuations may erroneously appear as noteworthy trends and, therefore, care must be exercised in interpreting the trends for these cancers. Multiple comparisons were adjusted to ensure an overall type I error rate of 0.05 through the joinpoint permutation test. However, spurious associations are still possible due to the large number of statistical tests conducted. Important sub-population trends may have been obscured, given the epidemiological heterogeneity of the study population, particularly its broad age range and ethnic diversity, and the potential for interaction of these with both the spectrum of cancers identified and health service utilisation. In addition, AYA cancers represent a heterogeneous group of diseases with specific etiologies. Nonetheless, the observed differences in the trends between the sexes and across cancer subgroups provide starting points for etiologic research. Testable hypotheses, including individual characteristics and environmental exposures, can be investigated in relation to specific cancers through further rigorous study designs.

## Conclusion

We have presented data representing a comprehensive longitudinal analysis of cancer incidence and mortality among AYAs in WA. Mortality from cancer showed persistent downward trends between 3% and 5% among young men and women in the last ten years of the study. We observed favourable trends in mortality, particularly among AYAs diagnosed with testicular cancer, HL, leukaemias and other neoplasms amenable to treatment. This might also reflect major trends in the risk factors and preventive interventions. Notwithstanding these major improvements, incidence continues to rise for certain cancer types such as ALL, HL, and breast, colorectal, testicular and thyroid cancers. Thus, further research into the aetiology of the distinctive spectrum of cancers affecting AYAs and more widespread adoption of screening, early diagnosis should become key priorities for cancer control among AYAs.

## Competing interests

The authors declare that they have no competing interests.

## Authors’ contributions

FAH conceived and designed the study, performed the analysis, interpreted the results and drafted the manuscript. DBP, GP, CDJH and KE critically reviewed the manuscript for important intellectual content and approved the final manuscript.

## Pre-publication history

The pre-publication history for this paper can be accessed here:

http://www.biomedcentral.com/1471-2407/12/151/prepub

## Supplementary Material

Additional file 1 Table S1. International Classification of Diseases for Oncology topography and histology codes (Online only). Click here for file
