# Supplementary material for: Cancer incidence and mortality trends in Australian adolescents and young adults, 1982–2007
Source: BMC Cancer. 2012 Apr 20;12:151. doi: 10.1186/1471-2407-12-151 (PMC3404933; doi:10.1186/1471-2407-12-151)
Supplement: Additional file 1 — Table S1. International Classification of Diseases for Oncology topography and histology codes (Online only). [file 1471-2407-12-151-S1.doc]

Table 1: International Classification of Diseases for Oncology topography and histology codes (**Online only**)

| **Diagnostic Group** | **Primary Site and ICDO−3 Histology** |
| --- | --- |
| **Leukemia**  ALL  AML & CML  Other | C000−C809; 9826−27,9835−37  C000−C809; 9840, 9861, 9866−67, 9871−74, 9891, 9895−97, 9910, 9920, 9863, 9875−76  C000−C809; 9742, 9800, 9801, 9805, 9820, 9823, 9831−34, 9860, 9870, 9930−31, 9940, 9945−46, 9948, 9963−64 |
| **Lymphoma**  NHL  HL | C000−C809; 9590, 9591, 9596, 9670, 9671, 9673, 9675, 9678−80  C000−C809; 9684, 9687, 9689, 9690, 9691, 9695, 9698−02, 9705, 9708, 9709, 9714, 9716−19, 9727−29  C000−C809; 9650−55, 9659, 9661−65, 9667 |
| **CNS** | C000−C809; 9410-11, 9420-21, 9424, 9401, 9440−42, 9400, 9381−84, 9423, 9430, 9450, 9451, 9460, 9391−94  C000−C722, C724−C809; 9380  C716; 9470−74  C000−C715, C717−C809; 9470−74  C000−C699, C730−C750, C754−C809; 9350−51, 9360−62, 9390, 9480, 9530−35, 9537−39, 9541, 9550, 9562, 9570  C700−C729, C751−C753; 9161, 9361−62, 9390, 9530−31, 9535, 9538, 9540, 9560, 9571  C700; 9532, 9534, 9537, 9539  C723, C753; 9360  C711; 9480, 9539  C713; 9480, 9533  C719; 9350  C714, C717; 9480  C709; 9539  C700−C729, C751−C753; 8000−05 |
| **Bone** | C000−C809; 9180−87, 9192−94  C000−C809; 9220−21, 9230−31, 9240, 9242−43  C000−C809; 9260, 9364−65  C000−C809; 8812, 9250, 9261, 9370−72  C400−C419; 8000−05, 8800−03, 8805−06, 9200 |
| **Soft Tissue** | C000−C809; 8810−11, 8813−15, 8820−24, 8830, 8832−33, 8835−36, 9252  C000−C809; 8900−04, 8910, 8912, 8920−21, 8991  C000−C809; 8804, 8825, 8840−97, 8982−83, 8990, 9040−44, 9120−39, 9141−50, 9170, 9251, 9561, 9580−81, 9970  C000−C699; C730−C750, C754−C809; 9540, 9560, 9571  C000−C809; 9140  C000−C399; C420−C809; 8800−03, 8805−06 |
| **Germ Cell**  Gonadal  Non−gonadal | C569,C620−C629; 9060−9065, 9070−9073, 9080−9085, 9090−9091, 9100−9102, 9105  C000−C568, C570−C619, C630−C699, C730−C750, C754−C809, C700−C729, C751−C753; 9060−9065, 9070−9073, 9080−9085, 9090−9091, 9100−9102, 9104−9105 |
| **Melanoma** | C000−C809; 8720−8723, 8726, 8728, 8730, 8740−8746, 8761, 8770−8774, 8780 |
| **Carcinoma**  Thyroid  Lip/mouth*  Gonads  Lung**  Breast  Cervix/Uterus  Colon/Rectum  Other | C739; 8010−89  C000−C109, C120−C148; 8010−89  C569, C620−C629; 8010−89; C000−C809; 8590−93  C330−C349; 8010−89  C500−C509; 8010−89  C530−C559; 8010−89  C180−C218; 8010−89  C110−C119, C300−C329, C760, C510−C529, C570−C579, C600−C619, C630−C639, C659, C669, C680−C689, C160−C169, C220−C221, C250−C259, C150−C159, C170−C179, C230−C249, C260−C269, C740−C749, C149, C219, C222−C229, C270−C299, C350−C439,C450−C499, C561, C568, C580−C599, C640−C648,C650−C658,C660−C668, C690−C738, C750−C759, C761−C809, C649, C670−C679; 8010−89  C809; 9010 |
| **Other** | C000−C809; 8959−60, 9490, 9500, 8963−64, 8970−73, 8981, 9363, 9501−23, 8680−11, 8600−50, 9000, 8670, 9013−15, 9054, 9731−41, 9743−64, 9766, 9769, 9960, 8930−51, 8980, 9020, 9050−53, 9110, 9160, 9270−30, 9950, 9962, 9980, 9982  C421; 9961, 9975, 9989  C000−C699, C730−C750, C754−C809; 9161  C000−C399, C420−C699, C730−C750, C754−C809; 8000−05 |
| **Total** |  |

CNS, central nervous system; ALL, acute lymphoblastic leukemia; AML and CML, acute and chronic myeloid leukemia; HD, Hodgkin’s disease; NHL, Non−Hodgkin’s lymphoma

M/F: Sex ratio

*Head and neck sites in lip, oral cavity, and pharynx

** Trachea, bronchus, and lung
